# Linear and nonlinear optical properties of DNA in suspensions

**DOI:** 10.1016/j.optmat.2025.117223

**Published:** 2025-06-10

**Authors:** Arturo Ramirez, Lipi Patel, Andres Morin, Jesus Poblano, Rebecca Sipen, Chhandak Basu, Anna Bezryadina

**Affiliations:** a Department of Physics & Astronomy, California State University Northridge, Northridge, CA, 91330, USA; b Department of Biology, California State University Northridge, Northridge, CA, 91330, USA

**Keywords:** DNA, Optical properties, Z-scan, Suspension, Laser exposure

## Abstract

Deoxyribonucleic acid (DNA) has the potential to serve as inexpensive, functionalized photonic material. Previous research has mostly focused on dry DNA thin films; however, little is known regarding the properties and photonics applications of DNA solvents. This paper compares the linear and nonlinear optical properties of different sized DNA in suspensions. In particular, small plasmid DNA, long lambda DNA, and extra-long strawberry DNA were studied. The linear optical properties were evaluated in terms of UV–Vis spectrophotometry, and the nonlinear optical properties were investigated using the closed-open continuous wave Z-scan technique. In addition, the paper investigated DNA suspension survivability to prolonged exposure to a 532 nm green laser. The results showed DNA structural integrity after the 532 nm laser exposure and optical characteristics comparable to synthetic polymers, indicating that DNA suspensions could be a good optical material for photonic devices and optoelectronics applications.

## 1. Introduction

Deoxyribonucleic acid (DNA), historically recognized as the carrier of genetic information, has recently attracted considerable attention as an innovative material for photonic and optoelectronic applications [1-3]. Due to its distinctive structural, chemical, and mechanical properties, DNA exhibits versatility both as a standalone biomaterial and as a hybrid structure with functional molecules [4-8]. Its robustness, thermal and photochemical stability, biodegradability, and tunable optical properties make DNA a compelling alternative to conventional synthetic polymers in photonics [9]. Moreover, DNA represents a cost-effective and readily accessible biopolymer. DNA can be isolated from animal waste, plants, or bacterial cultures and manipulated using well-established protocols, highlighting its potential as a sustainable material [10-12].

DNA-based materials in various formats and combination with different molecules—such as thin films, DNA–polymer hybrids, aqueous suspensions, and nanostructured constructs, offer exciting opportunities for a wide range of photonic applications including biosensing [13,14], nonlinear optics [15-17], and optoelectronic device integration [8,18]. DNA–CTMA thin films exhibit excellent film-forming ability, optical transparency in the visible range, and have shown promising results in nonlinear optical applications, such as saturable absorption and refractive index modulation [4,15]. DNA–polymer hybrids and DNA-doped materials have also demonstrated enhanced photostability and device performance, particularly when combined with dyes or nanoparticles [5,17,19]. Recent advances in DNA origami techniques have enabled the creation of thin films with intricate nanostructure designs at nanometer-scale precision. DNA’s unique base-pairing and programmability allow finely tuned photonic behavior, which has been employed in developing nanodevices, liquid crystal displays, biosensors, and other nanoscale sensing devices [13,18,20]. DNA hydrogels and DNA origami-based devices provide reconfigurable photonic structures and light manipulation capabilities under stimuli [21,22]. Furthermore, DNA has shown promise in developing new organic lasers, where DNA host matrix doped with gain dye can act as a biodegradable host for gain medium for a DNA-based distributed feedback laser [23].

However, despite these advances, a gap remains in understanding the optical properties of DNA in its unmodified, suspended form. Most prior work has focused on DNA films, where binding agents like CTMA modify the local environment of DNA [19,24]. These modifications may alter DNA’s intrinsic optical response and limit our understanding of its fundamental properties. Investigating the optical properties and responses of DNA suspensions can contribute to advancing optically active biomaterials. The suspensions of DNA, which possess a flexible distribution of density, can be manipulated through optical or mechanical means, enabling the creation of optical waveguides, sensors, liquid-based optical devices, and other nonlinear applications [13,20,25,26]. For the advancement of DNA application in optically active materials, optoelectronics, liquid-based photonic devices, and material manipulation, it is imperative to understand the fundamental optical properties of different DNA in suspensions, including both linear and nonlinear characteristics. Studies using DNA suspensions are scarce, and comparative studies across DNA types or molecular sizes are virtually nonexistent.

Linear absorption spectroscopy is widely used to assess DNA concentration and quality, offering insights into structural changes under various stress conditions [24,27-29]. The absorption spectra of DNA can vary significantly in response to UV exposure, extreme thermal treatment, and integration into hybrid structures, indicating that the photonic characteristics of DNA are sensitive to external stimuli [30,31]. Nonlinear optical properties, such as nonlinear absorption and refractive index, are often determined using techniques such as second harmonic generation and the Z-scan method. These have been employed to determine third-order nonlinearities in DNA–CTMA films and DNA–polymer hybrids [15-17] with reported values of the nonlinear refractive index in the order of 10^−13^ to 10^−8^ cm^2^/W, making DNA competitive with synthetic nonlinear optical materials like polydiacetylene or doped polymers [19,32,33]. However, most of these studies have been limited to thin films and chemically modified DNA and little is known about the nonlinear optical properties of pure DNA of different types in suspension and how DNA’s molecular size affects its nonlinear response.

In this study, we present a comprehensive analysis of the optical properties and stability of three different-sized DNA in suspension: pBR322 plasmid DNA, lambda DNA, and strawberry DNA. These samples represent a range of sizes and sources—plasmid, viral, and plant-based DNA—allowing us to examine how molecular weight and structural configuration impact optical behavior. We investigate their structural integrity and optical characteristics following exposure to a popular photonic laser wavelength (532 nm), in addition to UV and thermal treatments. The analysis of DNA suspensions revealed a significant capacity to withstand optical damage. We utilize spectrophotometry to quantify variations in linear absorption, apply gel electrophoresis to assess DNA denaturation, and employ the continuous wave (CW) Z-scan technique to evaluate the nonlinear absorption and nonlinear refractive index coefficients of each DNA type in suspension at a wavelength of 532 nm. This work provides evidence that DNA suspensions without surfactant modification can serve as a stable, biodegradable, and optically responsive material suitable for photonic applications.

## 2. Experimental methods

### 2.1. DNA sample preparation

In this study, we compared three different-sized DNA samples, namely, plasmid DNA (small), lambda DNA (medium), and strawberry DNA (large). All the DNA samples were either bought or isolated in the lab and stored in a TE buffer (10 mM Tris-HCl (pH 7.6) and 1 mM EDTA) in a −20 °C freezer. Frozen DNA was thawed at room temperature, stirred, and centrifuged before use in experimental procedures. All three DNA samples were diluted to 40 ng/μl using TE buffer in the optical experiments and scanned using a NanoDrop spectrophotometer to validate their purity by obtaining A260/A280 and A260/A230 ratios of approximately 1.8 and 2.0, respectively, and to ensure that the optical properties were not altered by unwanted precipitants [27].

#### 2.1.1. Plasmid DNA

Plasmids are small, circular, self-replicating DNA molecules present in prokaryotic cells. In this study, we used pBR322 plasmid DNA as the small-type DNA. The plasmid DNA is obtained from Thermo Scientific (Cat #SD0041) with a stock concentration of 0.5 μg/μl. It was one of the first and most common *E. coli* cloning vectors because of its small size (4361 base pairs) [10]. It is easy to handle and remains stable at room temperature for a long period of time.

#### 2.1.2. Lambda DNA

Lambda DNA is a well-known DNA vector acting as a molecular weight marker in gel electrophoresis (discussed later). It is a linear double-stranded DNA derived from an *E. coli* bacteriophage which served as our medium-sized DNA with 48,502 base pairs [10]. The lambda DNA was bought from New England Biolabs (Cat #N3011S) with a stock concentration of 500 μg/ml.

#### 2.1.3. Strawberry DNA extraction

We used genomic DNA from strawberry leaves as our longest DNA sample. It was isolated in the lab by using the modified CTAB protocol [11,12]. The CTAB buffer, which includes Tris, NaCl, EDTA, and CTAB was prepared in advance with polyvinylpyrrolidone (PVP) and 2-mercaptoethanol added right before extraction. Fresh strawberry leaves (20 mg) were ground using a mortar and pestle in the presence of liquid nitrogen, then mixed with 500 μl of CTAB buffer and incubated at 55 °C for an hour. Then, at room temperature, isoamyl: chloroform in the ratio of 24:1 was added, followed by centrifugation at 13000 RPM for 10 min for isolation of the supernatant. The supernatant was purified in a series of steps, beginning with a mixture of ammonium acetate and isopropanol, followed by 70 % ethanol, and finally, 95 % ethanol. The DNA pellet was obtained by drying the final pellet, then resuspending it in 100 μL of TE buffer and freezing it at −20 °C. To ensure quality control, all samples were tested using a Nanodrop 2000c spectrophotometer (Thermo Fisher Scientific, USA). Strawberry DNA is linear, double-stranded, and consists of hundreds of millions of base pairs. The CTAB extraction method, combined with cold temperatures, helps minimize DNA fragmentation but does not completely prevent it. As a result, the strawberry DNA suspension predominantly contains very large strands of DNA, spanning millions of base pairs, along with smaller fragments as short as 75 base pair.

### 2.2. Optical and thermal damage experimental procedures

Determining the damage threshold of DNA samples is critical for understanding their stability and resistance to laser irradiation, as DNA would be subjected to thermal and ionizing conditions. The DNA suspensions were subjected to damage assessments.

#### 2.2.1. Exposure to broad and focused beams

**Fig. 1.**
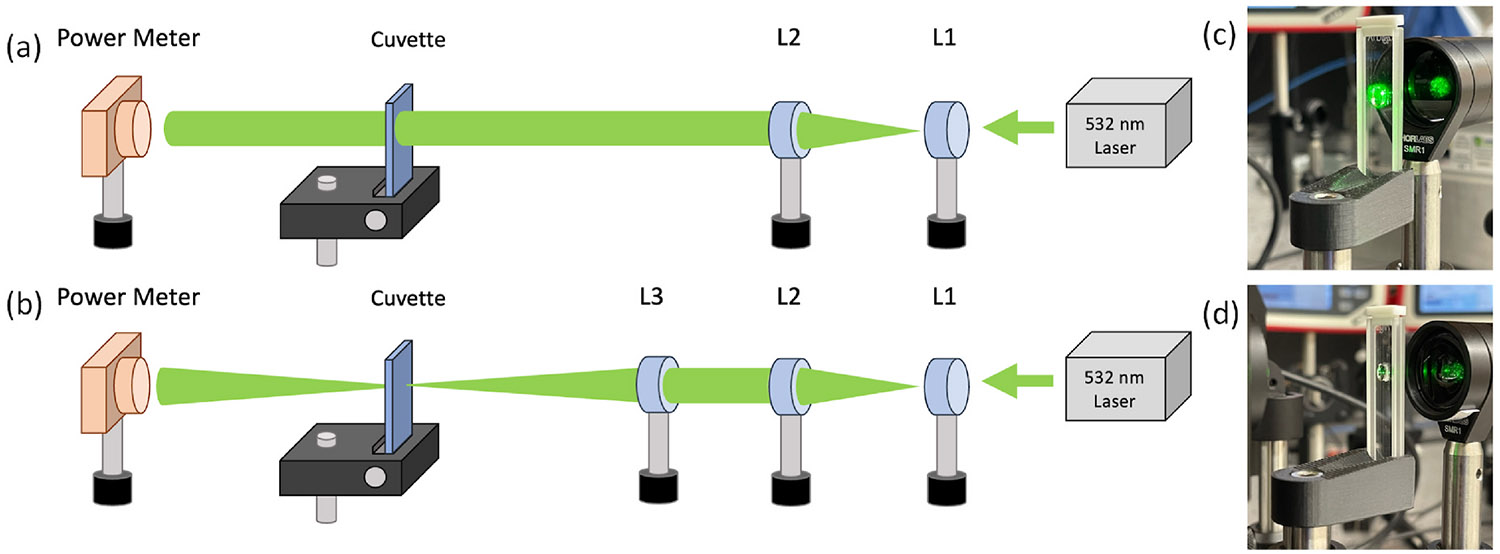
Experimental setups of broad beam illumination (a) and focused beam illumination (b); droplet of DNA sample is illuminated by broad beam (c) and focused beam (d).

Both the broad beam and focus beam optical setup used a laser light source of a 532 nm continuous wave diode-pumped solid-state laser (Msquared Sprout). In the broad beam illumination setup, the whole DNA sample was exposed entirely for 1 h to a strong laser beam as in Fig. 1(a-c). The 500 mW laser beam exiting the laser was expanded to 5.83 mm in diameter using lenses L1 and L2, resulting in an intensity of 3.75 W/cm^2^ at the sample. The illumination spot was slightly bigger than the droplet which is on average 4 mm in diameter.

To increase the power per area, the laser beam was focused on the droplet (Fig. 1(b-d)). In focused beam setup, in addition to the existing lenses L1 and L2 a 60 mm convex lens (L3) was used to reduce the laser spot size diameter to 6.97 μm at the focus point. This increased the intensity of laser beam illumination to 2.62 × 10^6^ W/cm^2^ at the sample. In the focused beam illumination experiments, only a small region of the sample was exposed for 1 h, but due to laser heating and convection inside a droplet, we may assume that majority of the DNA was exposed to radiation.

#### 2.2.2. Exposure to heat

Thermal effects were investigated because they are a likely byproduct of laser irradiation, and DNA is known to denature at temperatures around 95 ° C [34]. To ensure complete denaturation during heat exposure, DNA samples were sealed in 2 ml microcentrifuge tubes and heated on a GeneMate Digital Drybath at 95 ° C for 1 h and then quenched in an ice box and placed at −20° C to prevent renaturation.

#### 2.2.3. Exposure to UV light

A high enough energy transfer from the laser light can damage the DNA as the ionization potential of its molecules ejects electrons, causing physical deformation. Mutagenic effects of DNA are observed in the ultraviolet range of 230–320 nm with an ionization potential below 260 nm [35-37]. For our experiments, DNA samples were suspended in quartz Azzota cuvettes and exposed to UV radiation at 254 nm wavelength using a Philips TUV T8 illumination source with a transmitted power to the sample of 20 mW.

### 2.3. DNA spectroscopy

UV–Vis absorption spectra were collected using an Agilent Technologies Cary 60 UV–Vis spectrophotometer. Measurements were performed on three DNA samples and a TE buffer solution, with deionized water used as the blank. Spectra were recorded over a wavelength range of 200–700 nm with a data interval of 0.5 nm. The scan rate was set to 150 nm/min to ensure sufficient spectral resolution. Samples were measured in quartz cuvettes with a 1 cm path length at room temperature.

To assess the quality of the DNA samples and monitor changes in the linear optical properties of the DNA, a NanoDrop 2000c spectrophotometer was used, operating over a wavelength range of 220–350 nm [27]. To examine a sample, 1 *μ* L of DNA solution was pipetted carefully onto the measurement pedestal after being cleaned with nanopure water. TE buffer was used as the blank to help detect subtle changes in DNA properties and concentration. To compare samples across different conditions while accounting for concentration variations, all absorption spectra were normalized at 260 nm. Characteristic maximum absorption peaks for the DNA samples were identified at 257 nm preceded by a local minimum between 230 nm and 235 nm.

### 2.4. Gel electrophoresis analysis

Gel electrophoresis was used to determine denaturation of DNA after heat and light exposure. GeneRuler 1 kb Ladder (Thermo Scientific) was used to compare the approximate size of DNA fragments in the range of 75-25,000 base pairs. 1 % agarose gel was prepared using fresh 1X TAE buffer (Tris-acetate-EDTA) and 0.01 % (v/v) of SYBR safe dye (Thermo Fisher Scientific). The gel was run at 90 V for 45 min in 1X TAE buffer and 6X TriTrack DNA loading dye was used to visualize DNA bands under a blue light illuminator with orange filter.

### 2.5. Z-scan experimental setup and nonlinear optical characterization

Since its invention by M. Sheik-Bahae, the Z-scan has come to be a highly recognized method for studying the nonlinear optical properties of materials [33,38,39]. By using the closed-open Z-scan method, we can extract the nonlinear optical absorption coefficient (β) and the nonlinear index of refraction ($n_{2}$) of the three DNA types.

**Fig. 2.**
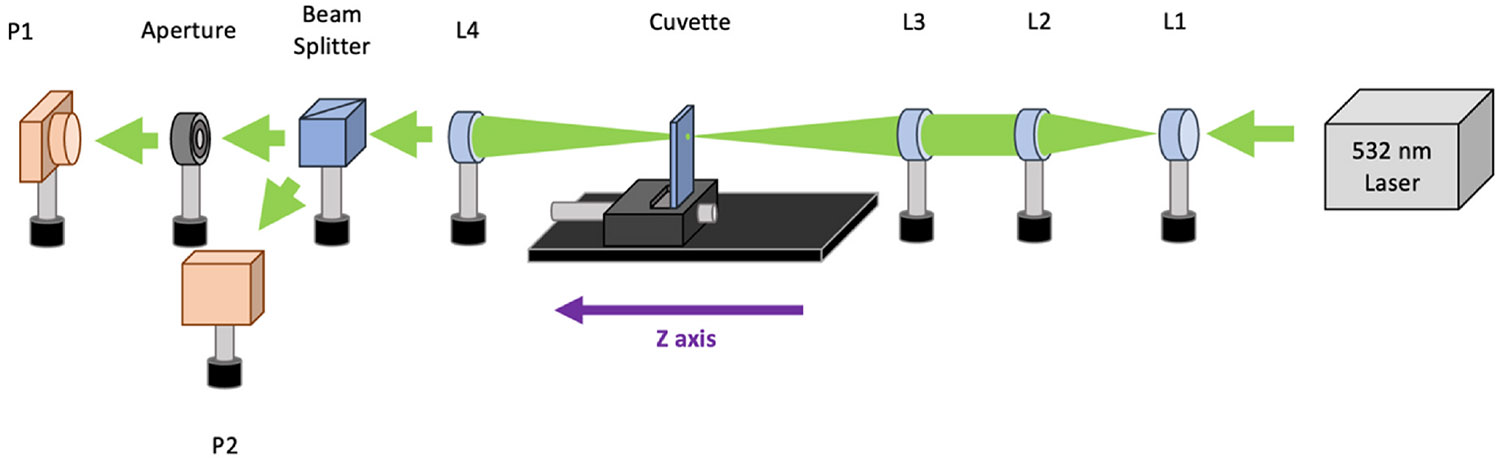
Optical setup for the Z-scan measurements with lenses L1, L2, L3, and L4 and photodetectors P1 and P2.

Fig. 2 shows the Z-scan experimental setup. A 1.00 W continuous wave 532 nm laser beam was expanded to 5.83 mm in diameter by using lenses L1 and L2 and focused by a 100 mm convex lens L3 to a spot size with a radius $w_{o}$ of 5.8 μm. As a result, the laser intensity at the focus was 1.89 × 10^6^ W/cm^2^ and the Rayleigh length $z_{o}$ was 0.20 mm. A DNA sample in a glass cuvette with a 1 mm path length (effective thickness) was translated along a 40 mm length by a motorized stage, with the beam waist located at 20 mm from the stage’s start. To minimize potential thermal effects due to continuous exposure in continuous-wave Z-scan, a beam shutter was incorporated into the setup to intermittently block the laser beam between measurements, preventing cumulative heating of the biosample and ensuring more accurate measurements of its nonlinear optical properties. For Z-scan experiments, DNA samples were diluted at a concentration of 100 ng/μl in TE buffer. Lens L4 collected and partially focused the exiting beam before splitting it 50:50 and capturing it with two Thorlabs photodetectors. Photodetector P1 detected a closed aperture signal in the presence of a partially closed aperture. The aperture had been closed by 75 %, resulting in a light transmission fraction of S = 0.36. Photodetector P2 detected an open aperture signal. The output power readings as a function of translation in the z direction allowed calculation of nonlinear coefficients. The nonlinear absorption of the sample can be determined using the open-aperture’s transmission power, while the nonlinear refraction can be extracted by analyzing the closed-aperture transmission signal.

## 3. Results & discussion

### 3.1. DNA survival after prolonged exposure to 532 nm green laser

#### 3.1.1. Linear optical absorption spectra results

**Fig. 3.**
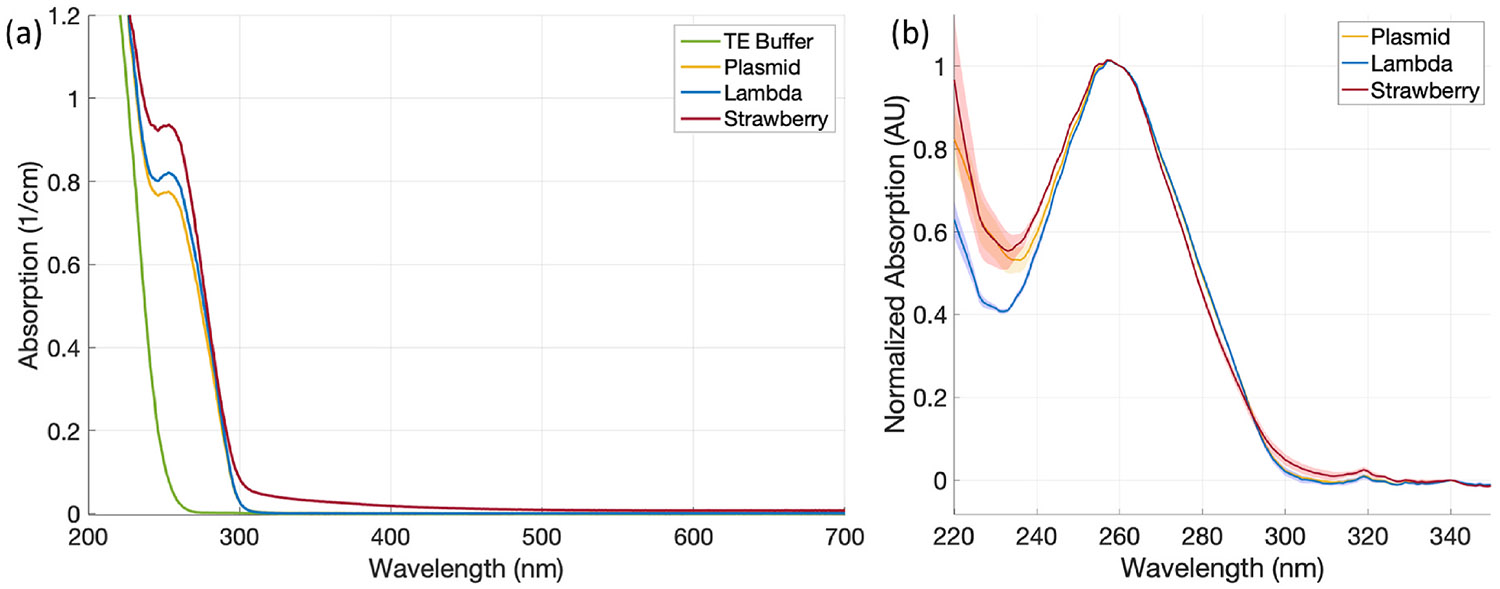
UV–Vis absorption spectra of TE buffer, plasmid DNA, lambda DNA, and strawberry DNA using deionized water as the reference blank (a), and normalized absorption spectra of the DNA samples relative to the TE buffer blank with normalization at 260 nm (b).

The absorption spectra of plasmid, lambda, and strawberry DNA samples were analyzed using both UV–Vis and NanoDrop spectrophotometers. Fig. 3(a) presents UV–Vis absorption spectra of TE buffer and the three DNA samples, using deionized water as a reference blank. The characteristic peak at 260 nm, which arises from UV absorbance of the purine and pyrimidine bases, is used to identify the presence of DNA within a sample [27,40]. The height of this absorption peak is proportional to the DNA concentration, effectively stretching the absorption curve vertically. In the measurements shown in Fig. 3(a), the concentrations were 40 ng/μL for plasmid DNA, 44 ng/μL for lambda DNA, and 51 ng/μL for strawberry DNA.

To detect subtle differences in DNA samples, additional absorption spectra were recorded using a NanoDrop spectrophotometer with TE buffer as the reference blank (Fig. 3(b)). The absorbance ratio at 260 nm and 280 nm (A260/A280) was used to evaluate DNA purity, with values above 1.8 indicating high-purity DNA. To minimize the effect of the slight variation in DNA concentration between samples, the average of at least 10 different absorption measurements normalized at 260 nm were taken for statistical purposes. By plotting the average of these spectra along with their error depicted by the shaded region, we can see how the samples change across the various conditions. Fig. 3(b) demonstrates the three DNA types in their pure original condition with slight variation in their spectra, centered in the 230 nm valley. DNA spectra have the same locations of peaks at 260 nm and valleys at 230 nm, since all samples are pure without contamination.

**Fig. 4.**
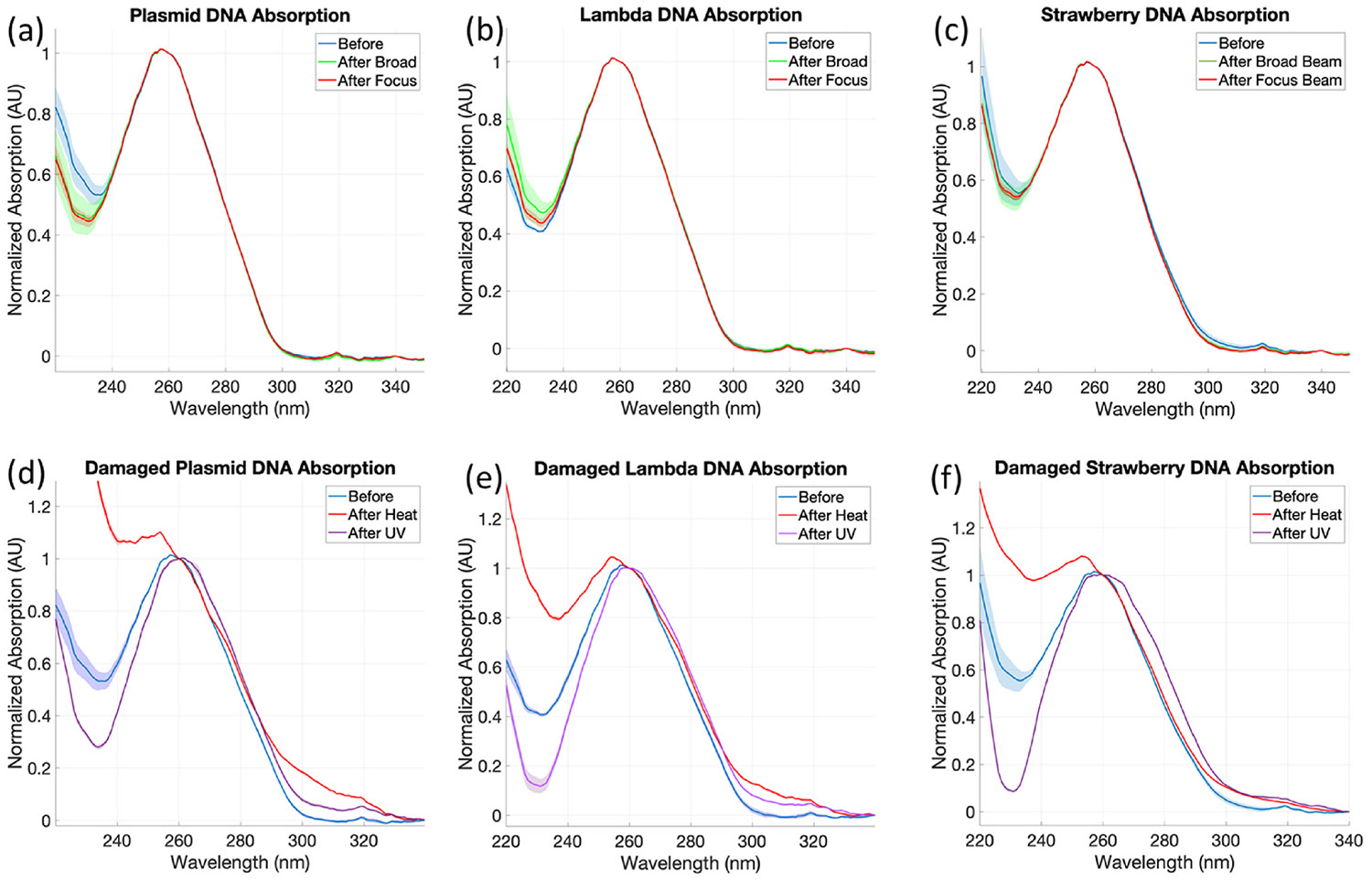
Changes in absorption spectra of plasmid, lambda and strawberry DNA samples after induced focused and broad beam laser exposure (a–c) and after induced heat and UV light exposure (d–f).

Fig. 4 illustrates the normalized absorption spectra of DNA samples before any treatments and afterward to exposure to a 532 nm laser, heat, and UV light. The absorption plots are normalized at 260 nm to eliminate effect of concentration fluctuations between samples. After an hour of exposure to high-power broad and focused laser beams, the absorption properties of DNA remain unchanged (Fig. 4(a-c)). The only observed changes are a variation in DNA concentrations by less than 10 % and slight shifts in the 230 nm valley. These changes are predominately due to the evaporation of the sample, which was minimized by keeping the sample in a tightly closed cuvette. Meanwhile, exposure to heat and UV light demonstrates a significant change in the absorption properties of the DNA samples (Fig. 4(d-f)). Heat exposure raises the valley around 230 nm with a less pronounced peak around 260 nm as DNA experiences denaturalization. UV exposure preserves the peak at 260 nm while lowering the valley at 230 nm, indicating the presence of DNA. These are expected results since UV light only damages DNA without destroying it or introducing any contamination. While the spectral signature of DNA exhibits only a slight change following UV exposure, major alterations in their structure will be evident through the analysis of DNA bands in agarose gel electrophoresis.

#### 3.1.2. Gel results

**Fig. 5.**
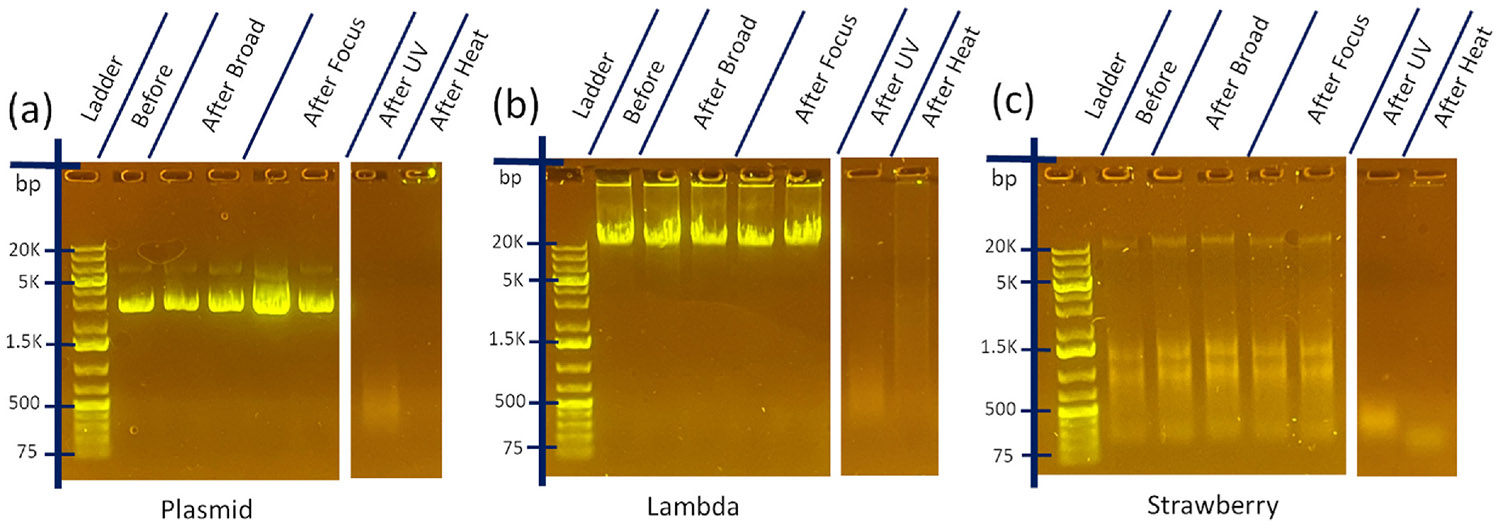
Agarose gel electrophoresis of plasmid DNA (a), lambda DNA (b) and strawberry DNA (c) before treatment, after induced broad and focused beam laser exposures, after UV light exposure, and after induced heat.

The gel electrophoresis analysis of the three DNA samples, both before and after their exposure to broad beam, focused beam, UV, and heat, is illustrated in Fig. 5. The DNA bands are compared to the ladder to determine their respective positions and structural integrity of DNA samples. The pBR322 plasmid DNA, with 4361 base pairs, is positioned beneath the 5 Kbp marker, while lambda DNA, with a length of 48.5 Kbp, exceeds the ladder’s 20 Kbp limit. Additionally, strawberry DNA, which comprises a mixture of different sizes, exhibits bands that are located above 20 Kbp and between 1.5 Kbp and 75 bp.

We can see that the three DNA samples exposed to both the broad beam and focused beam of the 532 nm laser demonstrate no observable change in the location of their bands in relation to the original DNA samples. The DNA bands all stop in the same location for plasmid, lambda, and strawberry DNA. The integrity of the DNA bands is most pronounced within the strawberry DNA as it has various bands of different sizes in reference to the DNA ladder that goes unchanged before and after laser exposure. In comparison, we can see an evident change in the location of the DNA bands, their near absence yet, after exposure to UV and heat. All three DNA types under exposure to UV change the location of their DNA band within the gel to a lower position in relation to the DNA ladder. The gel bands for the three DNA types after exposure to heat are also at a lower position but less visible indicating their degradation. Strawberry DNA exhibited the brightest DNA bands after exposure to both UV and heat demonstrating a stronger resilience to both UV and heat in comparison to the plasmid and lambda DNA.

Both spectral analysis and gel electrophoresis are in agreement, demonstrating no changes following 1 h of exposure to high power broad beam and focused beam. This indicates that DNA in suspensions maintains its integrity and stability when exposed to 532 nm laser illumination. The high optical damage threshold of DNA material is essential since high-power laser light intensity is needed to receive nonlinear optical responses in the material.

### 3.2. Nonlinear optical properties of DNA suspensions

**Fig. 6.**
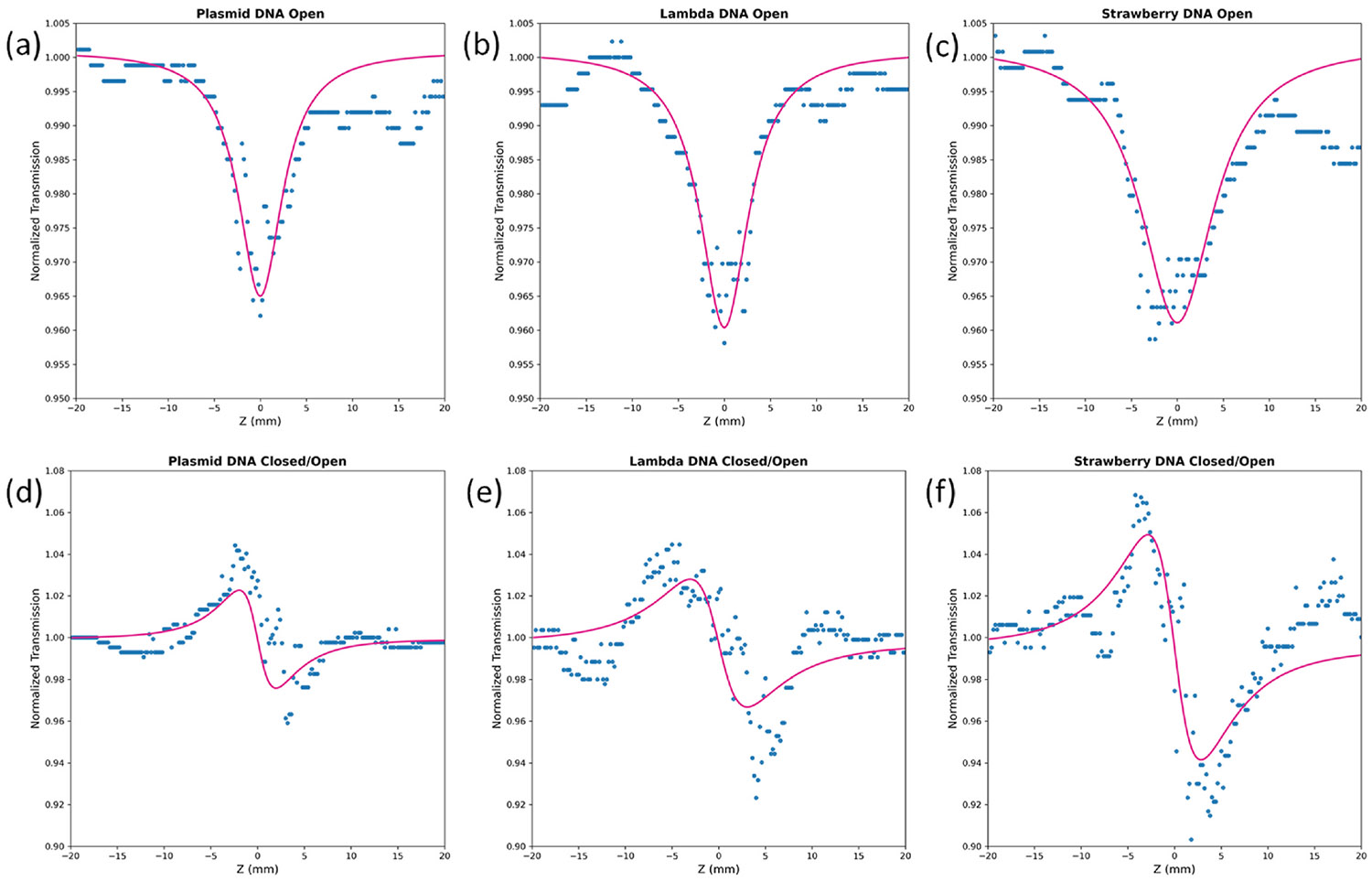
The normalized Z-scan data and fitting curves of plasmid, lambda and strawberry DNA samples (a–c) open aperture and (d–f) closed divided by open aperture data.

The nonlinear optical properties of DNA were measured by using the Z-scan method with a laser excitation wavelength of 532 nm. The normalized open-aperture Z-scan data of plasmid, lambda, and strawberry DNA samples are shown in Fig. 6(a-c). To determine the nonlinear absorption coefficient β, we implement a theoretical model for the normalized open-aperture transmission for two-photon absorption process, which can be expressed as [33]:

(1) $$T_{\mathrm{Open}\;\mathrm{Aperture}}=1-\frac{\beta I_{0}L_{\mathrm{eff}}}{2\sqrt{2}(1+z^{2}∕z_{0}^{2})}$$

where $I_{o}=1.89\times 10^{6}\;W∕{\mathrm{cm}}^{2}$ is the laser intensity at the focus, Leff=1 is the sample effective thickness, and $z_{o}$ is the Rayleigh length used as a fitting parameter. This analytical model has been widely adopted in prior studies utilizing continuous wave laser sources for Z-scan analysis [41-43].

The Pearson correlation coefficients for the fitted open-aperture transmittance curves are 0.88, 0.95, and 0.89 for plasmid, lambda, and strawberry DNA, respectively, indicating good agreement between experimental data and the theoretical model. The transmittance curves exhibit a characteristic valley at the focus point (z = 0), indicating presence of reverse saturated absorption in the DNA samples. Reverse saturated absorption is a nonlinear mechanism in which an increase in excitation intensity leads to a decrease in transmitted intensity. This effect results from two-photon absorption in DNA suspension [44].

Importantly, the TE buffer control exhibited no measurable nonlinear absorption under the same experimental conditions, with its nonlinear absorption coefficient β effectively near zero – comparable to that of water. In contrast, all three DNA samples demonstrated measurable nonlinear absorption, with β values on the order of 6 x10^−7^ cm/W, despite their near-zero linear absorption coefficients at 532 nm (see Table 1). Both the width and depth of the open-aperture valley increased slightly with the DNA size, suggesting a correlation between molecular size and nonlinear optical response. As summarized in Table 1, the β values exhibit minimal variation across the samples, with a slight increase observed with increasing DNA size.

Since nonlinear absorption is strong, the closed-aperture Z-scan measurements are asymmetric. To determine the nonlinear refractive index and estimate the closed aperture results in the absence of nonlinear absorption, the closed-aperture scan is divided by the open-aperture scan. The normalized closed over open aperture Z-scan data of plasmid, lambda, and strawberry DNA samples are shown in Fig. 6(d-f). The data for normalized closed over open aperture transmission are fitted using the following relationship [39,41,43,45,46]:

(2) $$T_{\mathrm{Closed}∕\mathrm{Open}\;\mathrm{Aperture}}=1-\frac{4\Delta \phi_{0}\;z∕z_{0}}{\left(9+z^{2}∕z_{0}^{2})(1+z^{2}∕z_{0}^{2}\right)}$$

where $\Delta \phi_{0}$ is the on-axis nonlinear phase shift of the laser beam. The Pearson correlation coefficients for the fitted closed-aperture Z-scan curves are 0.73, 0.73, and 0.82 for plasmid, lambda, and strawberry DNA, respectively, indicating a reasonable fit.

The normalized transmission curves have a peak followed by a valley shape. The peak-valley transmission indicates that DNA suspensions demonstrate a self-defocusing effect, with a negative nonlinear index of refraction. The light diverges as it propagates through the suspension, as focused light creates a local thermal lensing effect. Among the samples, strawberry DNA demonstrates the most pronounced transmission signature with the largest difference between peak and valley values, indicating a stronger nonlinear phase distortion, likely associated with larger DNA size.

The nonlinear index of refraction $n_{2}$ can be calculated from the phase shift $\Delta \phi_{0}$ extracted from the fitting curves using the relation [33]:

(3) $$n_{2}=\frac{\lambda \Delta \phi_{0}}{2\pi \;I_{0}\;L_{\mathrm{eff}}}$$

where λ is the wavelength of the laser. The extracted phase shift values, $\Delta \phi_{0}$ are 0.125, 0.151, and 0.266 for plasmid, lambda, and strawberry DNA, respectively. As shown in Table 1, the nonlinear index of refraction increases with the size of the DNA, with values ranging from approximately 5 x10^−12^ to 12 x10^−12^ cm^2^/W.

The nonlinear optical analysis of DNA suspensions revealed the nonlinear absorption coefficient, β, remains on the order of 10^−7^ cm/W, with only a slight increase with DNA size across the three DNA types studied—pBR322 plasmid DNA, lambda DNA, and strawberry DNA—despite their large differences in molecular size and origin. The measured β values are approximately three orders of magnitude larger than those reported for salmon-based DNA in deionized water measured by femtosecond Z-scan at 532 nm [4]. In contrast, the nonlinear refractive index, $n_{2}$ exhibits a clear increasing trend with DNA size, suggesting that DNA length and conformation influence refractive nonlinearities more strongly than the absorptive nonlinearity under CW excitation at 532 nm.

The measured $n_{2}$ values for pure DNA suspensions (5 x10^−12^ to 12 x10^−12^ cm^2^/W) are comparable to synthetic polymers with strong nonlinear optical properties, such as polydiacetylene paratoluene-sulfonate (PTS) with $n_{2}$ around 5 x 10^−12^ cm^2^/W in single crystal form [33,38]. Previous femtosecond Z-scan studies have reported $n_{2}$ ≈ 10^−13^ cm^2^/W for DNA–CTMA in butanol at 1030 nm [16] and $n_{2}$ ≈ 10^−14^ cm^2^/W for salmon DNA in deionized water at 532 nm [4]. Under CW Z-scan conditions, DNA–CTMA in butanol shows slow nonlinear responses with $n_{2}$ ≈ 10^−8^ cm^2^/W [16]. Our pure DNA suspensions in TE buffer at 532 nm exhibit $n_{2}$ values at the lower end of these CW Z-scan responses, suggesting that the presence of dye dopants and the CTMA matrix amplify DNA’s refractive nonlinearity. However, the similarity in magnitude supports the hypothesis that the nonlinear behavior originates, at least partially, from the DNA itself and not solely from the surrounding matrix or additives.

The increase in the nonlinear refractive index with DNA size is particularly notable. There are several possible mechanisms, which may contribute to the origin of nonlinear index refraction. Larger DNA molecules possess greater polarizability due to increased π-electron content, enhancing overall third-order susceptibility of the molecule [47]. Furthermore, the higher flexibility of long DNA strains may promote localized heating under laser illumination, which can cause local thermal lensing effects and localized refractive index changes due to temperature gradient. Although, in principle, light-induced orientation effects - where larger DNA strands align with optical gradients - could enhance nonlinear refractive effects, the high concentration of DNA in solution likely suppresses this mechanism due to molecular entanglement.

## 4. Conclusions

In this study, we have shown that DNA suspensions have the potential to be used as a biodegradable, non-toxic photonic material and an inexpensive alternative to synthetic materials.

We characterized the optical responses of three different-sized DNA suspensions, derived from plasmid (pBR322), viral (lambda), and plant (strawberry) sources. All three DNA samples possess stable and measurable nonlinear optical properties under continuous-wave laser exposure. The study found that all three types of DNA suspensions can withstand a focused 532 nm CW laser irradiation without any detectable changes in their properties. Gel electrophoresis and spectrophotometric analyses confirmed that the DNA samples preserved their structural integrity.

The ability of DNA to tolerate high laser power for a longer period of time demonstrates the high optical damage threshold of DNA, which is an essential parameter for nonlinear optics material applications. The evaluation of nonlinear optical properties showed that the nonlinear index of refraction changes with the molecular size of DNA, even if the amount of material remains the same, suggesting a size-dependent mechanism possibly linked to polarizability or conformational dynamics of longer DNA. However, the nonlinear absorption remains roughly the same regardless of DNA size. The nonlinear optical coefficients of DNA suspensions are comparable to those of conjugated polymers and inorganic compounds, highlighting DNA’s competitive performance in nonlinear optical modulation. All DNA samples exhibit a negative index of refraction, resulting in a self-defocusing effect for propagating light within the suspension, a characteristic commonly observed in biological samples.

The combination of our linear and nonlinear optical studies demonstrates the viability of these natural DNA extracts as photonic materials both due to their resilience to denaturation under optical exposure and their ability to exhibit relatively strong nonlinear optical properties. DNA suspension emerges as a robust polymer suspension with useful optical properties, making it a promising candidate for use in optically active biomaterials and photonics devices. As biodegradable, non-toxic, and renewable resources, DNA suspensions can serve as functional media for optical switching, where intensity-dependent refractive index changes are used to modulate light propagation. Additionally, their compatibility with aqueous environments and biological systems makes DNA suspensions ideal candidates for bio-integrated photonics, including flexible photonic circuits, liquid-based optoelectronics, microfluidic waveguides, and optofluidic biosensors.

## Figures and Tables

**Table 1:** The optical coefficients of plasmid, lambda, and strawberry DNA suspensions.

DNA type	α (1/cm)	β (x 10^−7^ cm/W)	$n_{2}$ (x 10^−12^ cm^2^/W)
Plasmid	0.0007	5.39	−5.2
Lambda	0.0009	6.08	−6.8
Strawberry	0.0065	6.12	−11.9

## Data Availability

Data will be made available on request.
